# Facile Synthesis of Nitrogen-Doped Microporous Carbon Spheres for High Performance Symmetric Supercapacitors

**DOI:** 10.1186/s11671-018-2713-0

**Published:** 2018-10-04

**Authors:** Zhongguan Liang, Hao Liu, Jianping Zeng, Jianfei Zhou, Hongjian Li, Hui Xia

**Affiliations:** 1 School of Physics and Electronics, Central South University, Changsha, 410083 China; 2 School of Physics and Electronics, Hunan University, Changsha, 410082 China

**Keywords:** Nitrogen-doped, Microporous carbon spheres, Supercapacitor, Energy storage and conversion, 81.05.Uw88.80.Fh82.47.Uv

## Abstract

Nitrogen-doped microporous carbon spheres (NMCSs) are successfully prepared via carbonization and KOH activation of phenol-formaldehyde resin polymer spheres synthesized by a facile and time-saving one-step hydrothermal strategy using triblock copolymer Pluronic F108 as a soft template under the Stöber-like method condition. The influence of the ethanol/water volume ratios and carbonation temperatures on the morphologies, pore structures and electrochemical performances of the prepared NMCSs are investigated systematically. The optimal NMCSs have a large specific surface area of 1517 m^2^ g^− 1^ with a pore volume of 0.8 cm^3^ g^− 1^. The X-ray photo-electron spectroscopy analysis reveals a suitable nitrogen-doped content of 2.6 at.%. The as-prepared NMCSs used as supercapacitor electrode materials exhibit an outstanding specific capacitance of 416 F g^− 1^ at a current density of 0.2 A g^− 1^, also it shows an excellent charge/discharge cycling stability with 96.9% capacitance retention after 10,000 cycles. The constructed symmetric supercapacitors using PVA/KOH as the gel electrolyte can deliver a specific capacitance of 60.6 F g^− 1^ at current density of 1 A g^− 1^. A maximum energy density of 21.5 Wh kg^− 1^ can be achieved at a power density of 800 W kg^− 1^, and the energy density still maintains 13.3 Wh kg^− 1^ even at a high power density of 16 kW kg^− 1^. The results suggest that this work can open up a facile and effective way to synthesize the NMCSs for electrode materials of high performance energy storage devices.

**Electronic supplementary material:**

The online version of this article (10.1186/s11671-018-2713-0) contains supplementary material, which is available to authorized users.

## Background

Energy security and global warming are facing serious challenges with increasing of the enormous depletion of traditional fossil fuel. The development of environment-friendly, green and sustainable energy storage devices with high energy and power output, and long life span are urgently needed [1]. Hence, in recent decades, supercapacitors have attracted considerable attention for a new generation energy storage devices due to its advantages of fast charge/discharge rate, high power density and excellent cycle stability [2–4]. Supercapacitors can be divided into electrical double-layer capacitors (EDLCs) and pseudocapacitors according to the charge storage mechanism. EDLCs, also known as carbon-based supercapacitors, have high power density and long cycle life arising from the reversible physical electrostatic charge accumulation at the electrode/electrolyte interface [5]. However, the electrochemical capacitance and energy density of EDLCs are still low because of the limited specific surface area, which severely hindered their commercialization [6]. On the contrary, pseudocapacitors possess higher energy density than EDLCs owing to the surface faradic redox reaction, but sacrifice the power density and the cycle life. Therefore, the most important in the development of supercapacitors is to increase their energy density without destroying its high power capability and long cycle stability.

In order to satisfy such a demand, a large number of multi-functional carbon materials in which combine the electrostatic adsorption mechanism with faradic redox reaction effect have been extensively designed and synthesized [7–11]. Among them, heteroatom-doped (especially nitrogen (N) and oxygen (O)) carbon spheres (CSs) as one of the most promising candidate due to the unique structural features (such as the regular geometry and good structural stability), stable physicochemical properties and advanced porosity [12–16]. Previous studies have revealed that heteroatom-doped was an effective strategy to optimize the properties of CSs, such as increasing the electronic conductivity, improving the surface wettability, and more important was to make additional contributions for capacitance enhancement through faradic reaction [13, 17].

Carbon precursors determine the final physical and chemical properties of the resulting carbon framework [18]. Phenolic resin, a three-dimensional network structured polymer, has become a fascinating precursors and widely used to synthesis the CSs due to the low cost, high thermal stability and easy transform to carbon materials [14, 19, 20]. In 2011, Liu et al. [21] firstly extended the Stöber method to synthesis resorcinol-formaldehyde resin polymer spheres and CSs with highly uniform and controllable size. Thereafter, a lot of Stöber-like methods have been developed and used to prepare N-doped CSs [22–24]. For examples, Lu and co-workers [25] have utilized the hexamethylenetetramine polymerize with resorcinol to fabricate the N-containing (1.21 at.%) ultramicroporous CSs under the Stöber condition. The obtained N-doped CSs as electrode materials for supercapacitors exhibited a high specific capacitance of 269 F g^− 1^ at 1.0 A g^− 1^. Tian et al. [26]. have successfully prepared the N-doped CSs with high nitrogen content from 5.5 wt% to 11.9 wt% by Stöber-like method that shown a good electrochemical capacitance of 127 F g^− 1^ at 10 mV s^− 1^. However, most of those Stöber-like synthetic methods generally required complicated procedures and/or a long processing time (usually more than 24 h), and many of these CSs exhibited a limited specific capacitance and unsatisfactory energy density. Therefore, it is a great challenge to developing a facile and rapid strategy to prepare N-doped CSs, which can satisfies the requirement for high performance supercapacitor applications.

Herein, we report a facile and time-saving one-pot hydrothermal synthesis method to prepare N-doped microporous carbon spheres (NMCSs) for high performance supercapacitor electrode materials. Phenol-formaldehyde (PF) resin spheres are polymerized by one-step hydrothermal reaction of phenol and formaldehyde under the extension of Stöber method condition, in which the triblock copolymer (Pluronic F108, PEO_132_-PPO_50_-PEO_132_) is used as soft-template and the ammonium hydroxide is used as catalytic agent and nitrogen source. The whole hydrothermal synthesis time can be remarkably reduced compared with the Stöber-like method in previous reported. The NMCSs with large surface area and suitable nitrogen content are successfully obtained via the carbonization and KOH chemical activation of PF resin spheres. As a result, the prepared NMCSs as electrode materials for supercapacitor exhibit an outstanding specific capacitance of 416 F g^− 1^ at a current density of 0.2 A g^− 1^ and excellent cycling stability with 96.9% capacitance retention after 10,000 charge/ discharge cycles. Moreover, the constructed symmetric supercapacitor devices (SSDs) can deliver a high energy density of 21.5 Wh kg^− 1^. The results indicate that the synthesized NMCSs are promising electrode materials for high performance supercapacitors.

## Methods

### Materials

Phenol, formaldehyde (37 wt%), ammonia solution (25 wt%), anhydrous ethanol, polyvinyl alcohol (PVA) and KOH were analytical reagent purchased from Sinopharm Chemical Reagent Co. Ltd. Triblock copolymer Pluronic F108 (Mw = 14,600, PEO_132_-PPO_50_-PEO_132_) and polyte-trafluoroethylene (PTFE, 60 wt%) were purchased from Aladdin. All chemicals and reagents were as received without further purification before used.

### Synthesis of NMCSs

The NMCSs were synthesized by the modified extension of Stöber method [21]. In a typical synthesis, 0.5 g F108 was firstly dissolved in 80 mL mixture solvent (the volume ratio of ethanol/deionized water was 4.3:1, and other ratios of 7:1, 3:1 and 1:1 were used for comparison) stirring at room temperature for 10 min to form clear solution. Then, 3 mL ammonia solution, 1.2 g phenol and 4.5 mL formaldehyde were added into the above system and continue stirring for 30 min. After that, the resulting solution was transferred to a sealed 100 mL Teflon-lined stainless steel autoclave and followed by hydrothermal reaction at 170 °C for 6 h to fabricate PF resin polymer spheres. The obtained pale-yellow precipitates were rinsed by deionized water and anhydrous ethanol for several times, and then dried at 80 °C for 12 h. After collection, the products were annealed at different carbonization temperatures (500 °C, 600 °C, 700 °C or 800 °C) for 3 h and followed by KOH activated in mass ratio of 1:2 at 700 °C for 1 h under N_2_ flow to fabricate the NMCSs (denoted as NMCSs-x, herein x represents the carbonization temperature).

### Characterization

The morphologies of the NMCSs were characterized by scanning electron microscopy (SEM, Nova NanoSEM230). Transmission electron microscopy (TEM) was investigated with a Tecnai G2 F20 S-TWIX instrument. X-ray diffraction (XRD) patterns were carried out with a SIEMENS D500 diffractometer with Cu Kα radiation (*λ* = 0.15056 nm). X-ray photo-electron spectroscopy (XPS) measurements were conducted on an ESCALAB 250Xi instrument with Al Kα radiation. The N_2_ adsorption-desorption isotherms were measured at 77 K with an ASAP 2020 instrument. The Brunauer-Emmet-Teller (BET) and Barret-Joyner-Halenda (BJH) methods were used to calculate the specific surface area and the pore size distributions of the materials, respectively.

### Electrochemical Measurement

All the electrochemical measurements were performed on an electrochemical workstation (CHI660E, Shanghai Chenhua Instruments). The working electrodes were prepared by the mixing of the NMCSs active materials, PTFE and acetylene black with a mass proportion of 80:10:10 in ethanol. The mixing materials were coated on the nickel foam, and the mass of the active materials in each piece working electrode was about 3 mg cm^− 2^. The electrochemical performances of the NMCSs electrodes were characterized by cyclic voltammetry (CV), galvanostatic charge/discharge (GCD) and electrochemical impedance spectroscopes (EIS) measurements with a classical three-electrode system in 6 M KOH electrolyte solution using platinum foil and Hg/HgO as the counter electrode and reference electrode, respectively.

The SSDs were assembled by the NMCSs-600 electrodes and the gel electrolyte of PVA/KOH. A modified method was used to prepared the PVA/KOH gel electrolyte [27]. Typically, 2 g PVA was dissolved in 12 mL deionized water at 80 °C under stirring until the solution became clear. After that, 1.5 g KOH was dissolved in 3 mL deionized water, and was dropwise added into the above system. The mixture solution was further stirring for 30 min at 80 °C, and then cooled down to room temperature. Two identical NMCSs-600 electrodes made by the above method were immersed in the PVA/KOH gel solution for 5 min, and over laying the two NMCSs-600 electrodes face-to-face which were separated by a membrane. After the gel solidified under room temperature, a SSD was successfully prepared, but without encapsulation (as shown in Additional file 1: Figure S1).

The gravimetric specific capacitance, energy density and power density were calculated from discharge curves according to the following equations:

1 $$ Cg=\frac{I\Delta t}{m\Delta V} $$

2 $$ Cs=\frac{I\Delta t}{M\Delta V} $$

3 $$ E=\frac{Cs\Delta {V}^2}{2\times 3.6} $$

4 $$ P=\frac{3600E}{\Delta t} $$

where *I* (A) is the charge/discharge current, Δ*t* (s) is the discharge time, Δ*V* (V) is the potential window, *m* (g) is the active material mass of the NMCSs electrodes, *M* (g) is the total active material mass of the NMCSs-600-based SSD, *C*_*g*_ (F g^− 1^) is the specific capacitance of the NMCSs electrodes, *C*_*s*_ (F g^− 1^), *E* (Wh kg^− 1^) and *P* (W kg^− 1^) are the specific capacitance, energy density and power density of the NMCSs-600-based SSD, respectively.

## Results and Discussion

### Fabrication of NMCSs

**Scheme 1:**
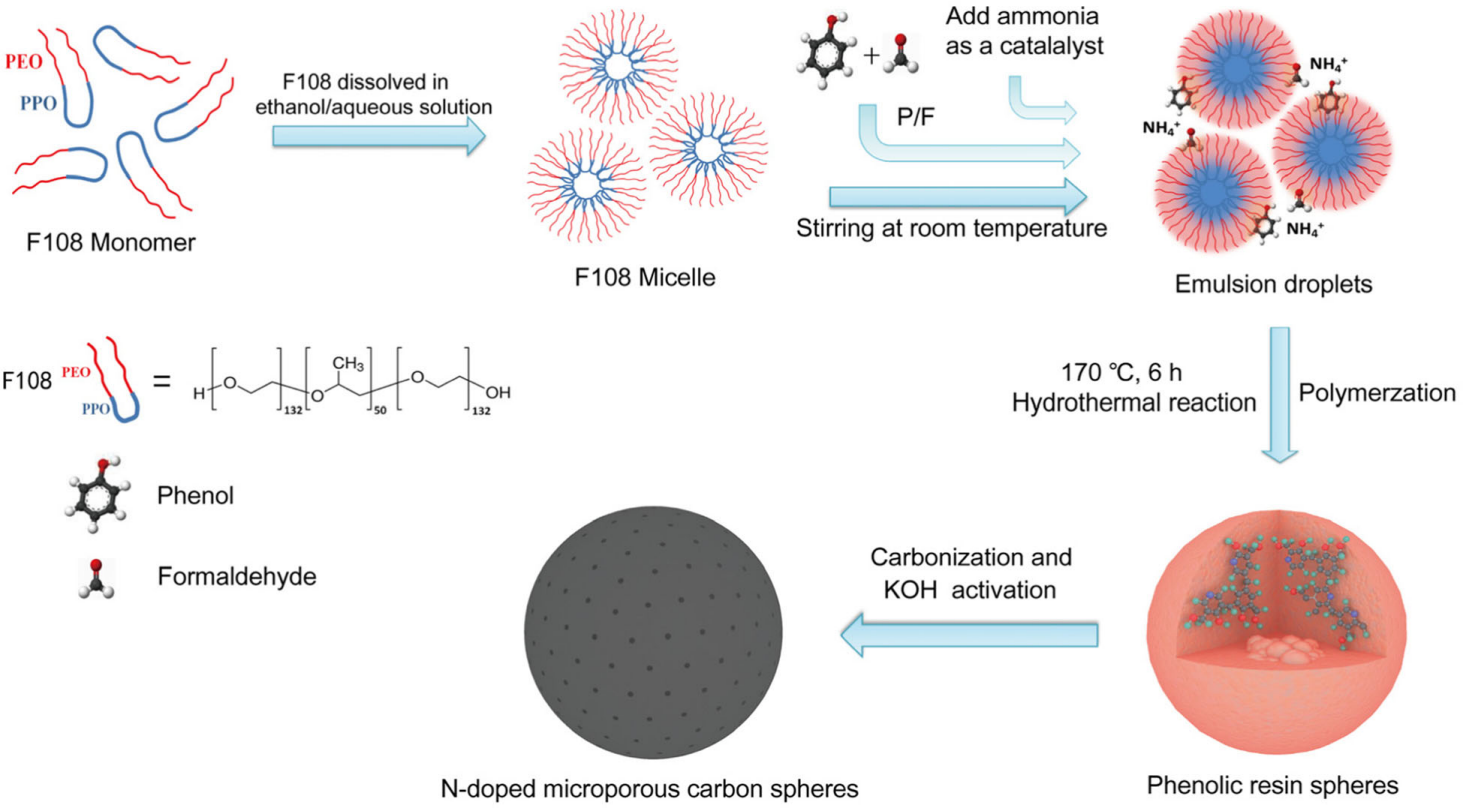
Schematic illustration of the fabrication process of NMCSs

The synthesis route was illustrated in scheme 1. The triblock copolymer Pluronic F108 with a big hydrophilic/hydrophobic ratio was used as a soft-template, ethanol and deionized water were involved as co-solvents, phenol and formaldehyde were selected as carbon precursors. The Pluronic F108 monomers was firstly dissolved in ethanol/water solution to form F108 micelles as a structure-directing and pore-forming agent [28]. Then, the emulsion droplets were formed through the hydrogen bonding interaction between PF precursors, of which with many hydroxyl groups (-OH), and PEO chains of F108 [29, 30]. During the process of hydrothermal reaction (a typical temperature was 170 °C), emulsions were further cross-linking polymerized to synthesis PF resin polymer spheres under the catalysis of NH_4_^+^ [21]. It was noteworthy that the reaction time was extremely short (just take 6 h) because of the high concentration of ammonia and high hydrothermal temperature accelerating the polymerization process. However, the yields of the production were reduced with further shorten the reaction time. Finally, the NMCSs were obtained via carbonization and KOH activation of PF resin spheres.

### Morphology and Structure

**Fig. 1:**
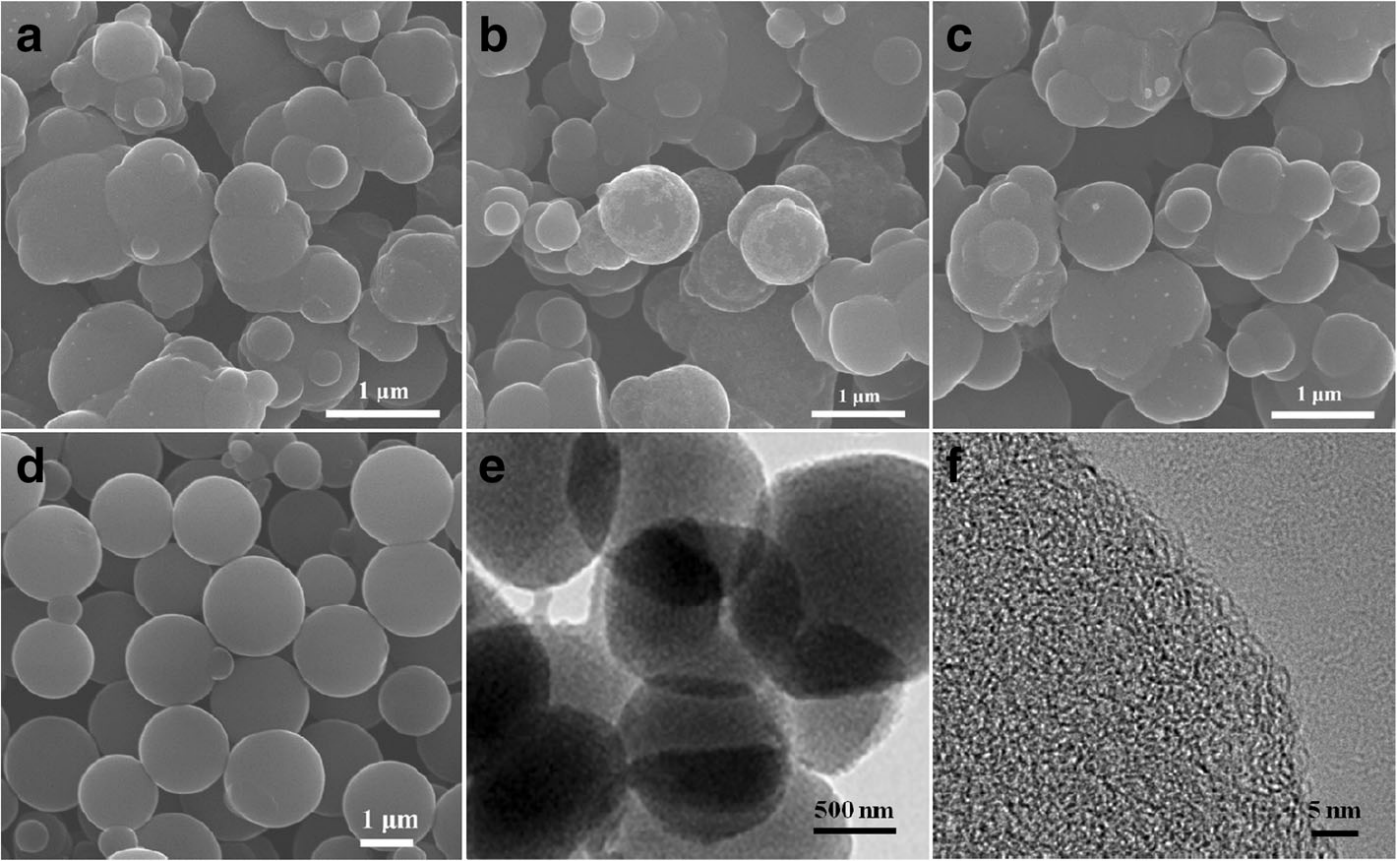
SEM images of the NMCSs samples synthesized at different ethanol/water volume ratios of (**a**) 7:1, (**b**) 4.3:1, (**c**) 3:1 and (**d**) 1:1, (**e**) TEM and (**f**) HR-TEM images of the NMCSs-600

Figure 1 a ~ d show the SEM images of the synthesized NMCSs at different ethanol/water volume ratios. It is indicated that the NMCSs have regular spherical particles but encountered agglomeration at high volume ratios of 7:1, 4.3:1 and 3:1, respectively. As shown in Fig. 1d, when the ethanol/water volume ratio is 1:1, the NMCSs have smooth surface, perfect spherical morphology and good dispersity, and the diameter of CSs are mainly concentrated in 1.2 to 2 μm. It can be seen that the spherical degree and dispersity of NMCSs are gradually getting better with the decreases of ethanol/water ratio. By increasing the quotient of water, the surface tension decreases [31], may lead to the lower cross-linking density of adjacent phenolic resin. Therefore, the PF resin polymer spheres with well dispersity and smooth surface are formed when decreasing the volume ratio of ethanol/water. The TEM image of NMCSs-600 (Fig. 1e) presents the spheres morphology. The HR-TEM image (Fig. 1f) shows clear microporous structure which provides sufficient active site and more efficient paths for a high specific capacitance.

**Fig. 2:**
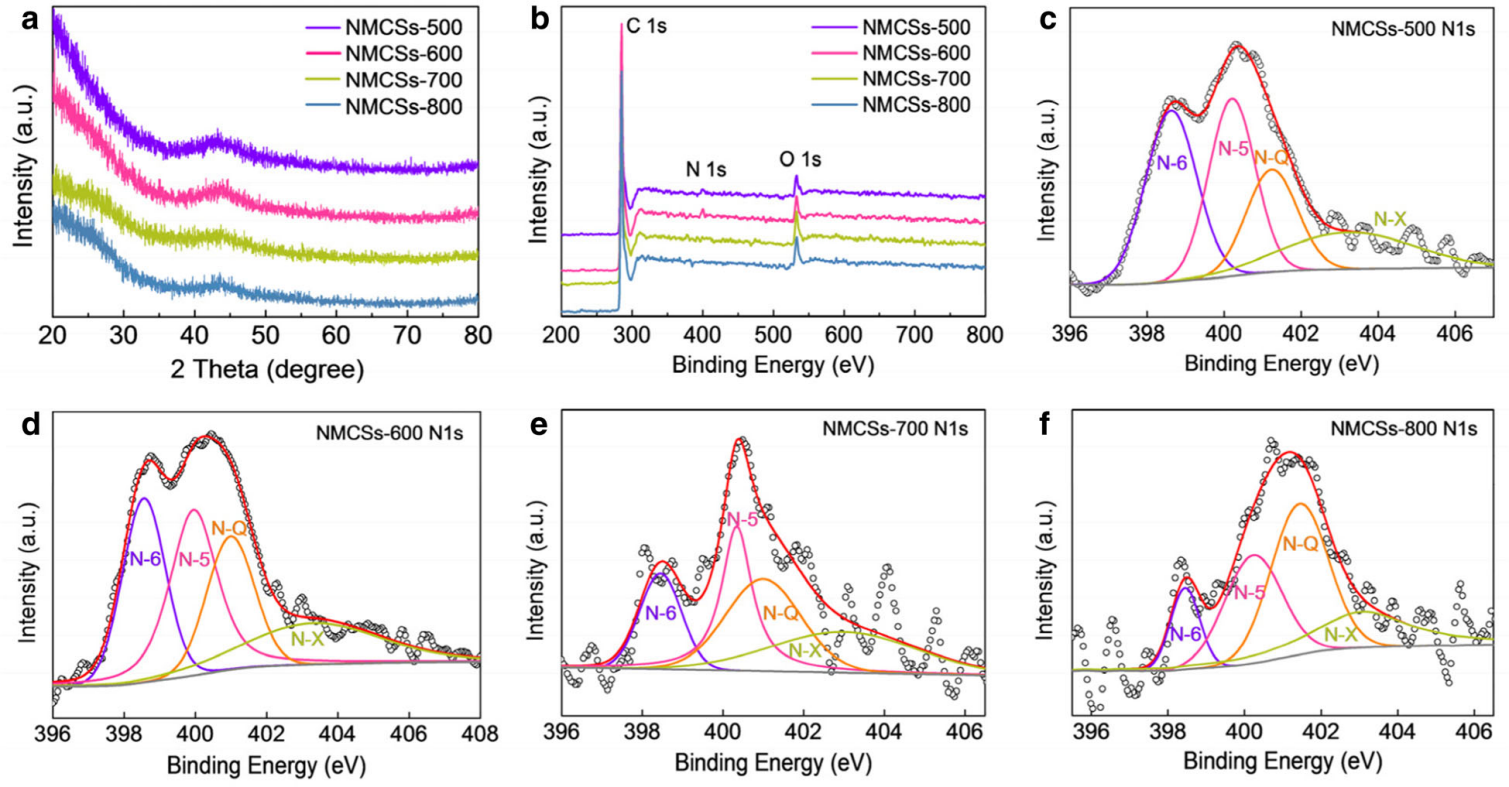
(**a**) XRD patterns and (**b**) XPS survey spectra of the as-prepared NMCSs materials, and the high-resolution N 1 s spectra at different carbonization temperatures of (**c**) 500 °C, (**d**) 600 °C, (**e**) 700 °C and (**f**) 800 °C

Fig. 2a presents the XRD patterns of NMCSs samples at different carbonization temperatures. One obvious broad diffraction peak located at ca. 2*θ* = 44°, the other one at ca. 2*θ* = 25° is gradually formed with the increases of carbonization temperature. These two peaks corresponding to the (100) and (002) lattice planes respectively, indicate that the as-prepared NMCSs are amorphous carbons.

### Composition Analysis

In the extension of Stöber method, ammonia aqueous plays an important role for the preparation of the PF resin spheres. Not only act as a catalyst to initiate the polymerization of PF resin, but also serve as a nitrogen source to introduce the N heteroatom into carbon frameworks [25]. Therefore, the chemical compositions of the prepared materials are explored by XPS measurement. Figure 2b shows the XPS surveys of NMCSs materials at different carbonization temperatures. Three obvious peaks of C 1 s, N 1 s and O 1 s are located at binding energy of 284.8 eV, 400.5 eV and 532.9 eV, respectively. It is manifest that the N and O heteroatoms have been successfully doped into the CSs matrix, which is consistent with the other previous research results [22]. The XPS elemental compositions analyses of NMCSs are show in Table 1. It reveals that the NMCSs-600 has the highest N relative content of 2.6 at.%. However, with the carbonization temperature increase to 800 °C, the content of N decreases to 0.9 at.%. This should be explained by the decomposition and conversion of N-containing functional groups at high temperature [15]. The high resolution N 1 s spectra of NMCSs materials at different carbonization temperatures are shown in Fig. 2c ~ f. Four characteristic peaks are located at binding energy of 398.5 eV, 400.2 eV, 401.0 eV and 403.2 eV, which are corresponding to pyridinic-N (N-6), pyrrolic-N (N-5), quaternary-N (N-Q) and pyridine-N-oxides (N-X) respectively. Table 1 gives the relative ratios of N-6, N-5, N-Q and N-X to the total N 1 s in the corresponding NMCSs. The ratio of N-6 undergoes a striking decrease from 32.4% to 10.7% as the carbonization temperature raise from 500 °C to 800 °C. The NMCSs-600 material has the highest N-5 ratio of 31.7%, but followed by reduce with further increase the carbonization temperature. On the contrary, the ratio of N-Q undergoes a sharp increase from 19.4% to 38.5% as the carbonization temperature increase, which is similar to the other carbon materials [9]. Each chemical state of N has different effects on electrochemical performances of supercapacitors. Studies have revealed that the negatively charged N-6 and N-5 were identified as electrochemically active and electron donors and thus contribute to pseudocapacitance reaction, while the positive charged N-Q and N-X were mainly to improve the charge transfer and enhance the electric conductivity of carbon materials [22, 25]. So, it is reasonable to infer that the NMCSs-500 and NMCSs-600 will show a larger pseudocapacity, while the NMCSs-700 and NMCSs-800 will show a better electrical conductivity. The high resolution C 1 s spectra of NMCSs samples (Additional file 1: Figure S2) show three characteristic peaks are located at 284.7 eV, 285.4 eV and 288.6 eV, which can be assigned to C=C, C–OH and C–N environments respectively [32]. The C–N peak also reflects the N-Q environment in the N 1 s spectra. In addition, the high resolution spectra of O 1 s (Additional file 1: Figure S3) can be deconvoluted into three individual peaks which are located at binding energy of 531.3 eV, 533.3 eV and 536.4 eV, corresponding to C=O, C–OH and COOH, respectively [7]. Generally, the existence of O-containing groups can not only benefit to additional pseudocapacitance that thanks to the redox reaction of electron donors, but also can enhance the wettability of the materials surface via the formation of polar functional groups. These results confirm that the N- and O-doped CSs are successfully synthesized.

**Table 1:** XPS for the elemental composition analyses of NMCSs and the relative ratios of nitrogen species to the total N 1 s

Samples	C (at.%)	O (at.%)	N (at.%)	N-6^a^ (398.5 eV)	N-5^b^ (400.2 eV)	N-Q^c^ (401.0 eV)	N-X^d^ (403.2 eV)
NMCSs −500	92.1	5.8	2.1	32.4%	30.4%	19.4%	17.8%
NMCSs −600	92.5	4.9	2.6	26.4%	31.7%	21.1%	20.8%
NMCSs − 700	91.2	7.7	1.2	17.3%	27.2%	30.3%	25.2%
NMCSs −800	92.8	6.3	0.9	10.7%	27.8%	38.5%	23.0%

^a^pyridinic-N

^b^pyrrolic-N

^c^quaternary-N

^d^pyridine-N-oxides

### Nitrogen Adsorption Studies

**Fig. 3:**
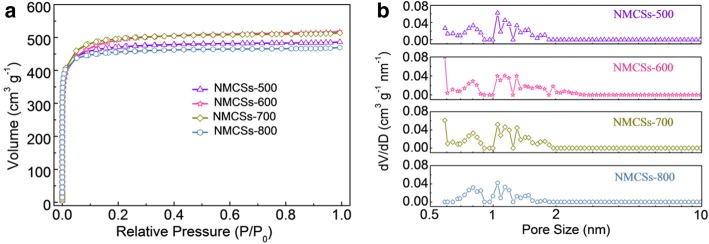
(**a**) Nitrogen adsorption/desorption isotherms and (**b**) pore size distribution curves of NMCSs materials

Nitrogen adsorption/desorpotion isotherms of NMCSs are presented in Fig. 3a. All of the resultant NMCSs delivered the typical type I isotherms with a steep uptakes at low relative pressures of P/P_0_ < 0.05, illustrating abundant micropores [33, 34]. A high N_2_ adsorption horizontal plateau at relative pressures of 0.1 < P/P_0_ < 1 means that has high specific surface area and larger pore volume. The pore size distribution curves of NMCSs are shown in Fig. 3b. It can be seen that plenty of micropores are concentrated in the range of 0.7 ~ 2 nm. The micropores of NMCSs can be attributed to the decomposition of F108 and PF resin polymers during the high temperature carbonization process and the chemical activity of KOH [23, 28]. Table 2 summarizes the specific surface area and the pore structure parameters of NMCSs. The total pore volume enlarges with increasing the carbonization temperature from 500 °C to 600 °C. As well as the specific surface area are increasing with the pore volume simultaneously. The results indicate that an increase of pore volume favors the increases of the specific surface area. The NMCSs-600 has the highest specific surface area of 1517 m^2^ g^− 1^ with the largest total pore volume of 0.8 cm^3^ g^− 1^, which offers enough electrode/electrolyte contact interface and abundant active sites for electrical double layer and benefits to enhance the electrochemical performances. When the carbonization temperature further rises to 800 °C, however, both the total pore volume and specific surface area are decreased noteworthy, which may due to the collapse or/and shrink of pores [7, 8]. Furthermore, there are small quantity mesoporous volumes, which arise from the stacking of CSs. Thus, it can be concluded that the carbonization temperature has a significant influence on the control of pore structure for NMCSs. The above structure characterizations and analyses mean that the NMCSs samples, especially NMCSs-600, may have excellent electrochemical performance as electrodes materials for EDLCs.

**Table 2:** Adsorption parameters of NMCSs obtained by analysis of the nitrogen adsorption isotherms and corresponding pore size distributions

Samples	*S*_BET_^a^ (m^2^ g^− 1^)	*V*_total_^b^ (m^3^ g^− 1^)	*V*_micro_^c^ (m^3^ g^− 1^)	*V*_mesol_^d^ (m^3^ g^− 1^)
NMCSs-500	1436	0.75	0.64	0.11
NMCSs-600	1517	0.80	0.56	0.24
NMCSs-700	1515	0.79	0.66	0.13
NMCSs-800	1384	0.73	0.64	0.09

^a^Specific surface area calculated by BET method

^b^Total pore volume, ^c^Micropore volume, ^d^Mesopore volume

### Electrochemical Performance of the NMCSs Electrodes

**Fig. 4:**
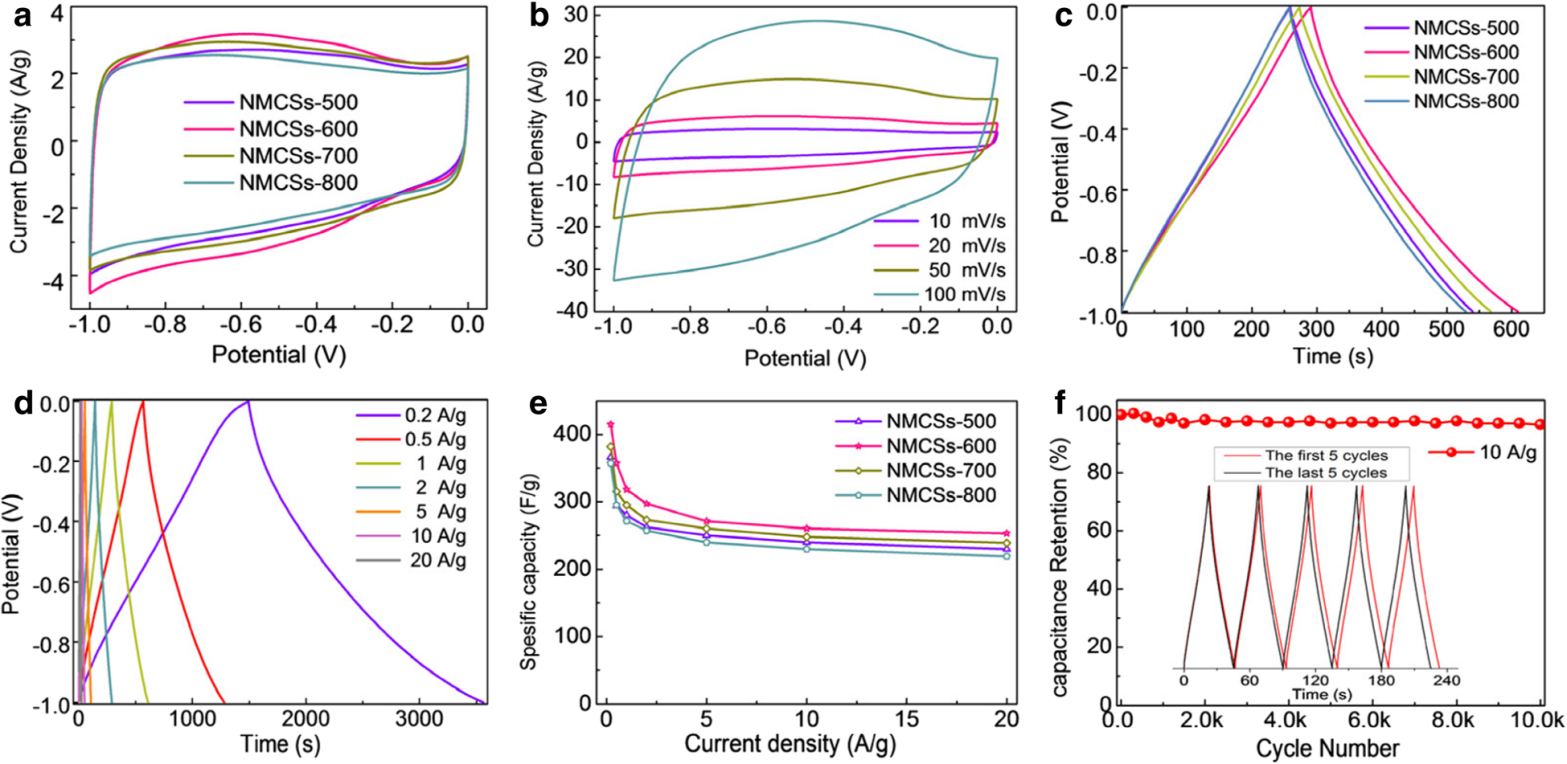
(**a**) CV curves of NMCSs electrodes at scan rate of 10 mV s^− 1^, (**b**) CV curves of the NMCSs-600 electrode at different scan rates from 10 to 100 mV s^− 1^, (**c**) GCD curves of NMCSs electrodes at current density of 1 A g^− 1^, (**d**) GCD curves of the NMCSs-600 electrode at different current densities, (**e**) Specific capacitance of NMCSs electrodes as a function of current densities, and (**f**) Cycling performance of the NMCSs-600 electrode at current density of 10 A g^− 1^ for 10,000 cycles and the inset shows the GCD curves of the first five and last five cycles, with a three-electrodes system in 6 M KOH aqueous solution

To evaluate the electrochemical performances of the obtained NMCSs as electrode materials for supercapacitors, the CV, GCD and EIS are carried out with a three electrode system in 6 M KOH aqueous electrolyte. Figure 4a shows the CV curves of NMCSs, all samples exhibit symmetrical quasi-rectangular shapes at a scan rate of 10 mV s^− 1^. It should be noticed that the obvious reversible humps, attributed to the redox reaction caused by N- and O-doped, are demonstrated in the potential window of − 0.8 to − 0.2 V. The NMCSs-600 material has the most prominent hump because of the highest N-doped concentration and moderate O-containing, which is corresponding to the previous XPS analysis. This result reveals that the N- and O-containing functional groups can contribute to the occurrence of the Faradaic reaction. Furthermore, the NMCSs-600 has a higher current density than other samples due to the high specific surface area and high N-doped concentration, which can give rise to an enhancement of specific capacitance. The CV curves of NMCSs-600 electrode at different scan rates are shown in Fig. 4b. It can be seen that the quasi-rectangular shape can be maintained even at a high scan rate of 100 mV s^− 1^. It indicates that the NMCSs-600 material has excellent rate capability, which is attributed to the unique porous spherical structure generate the short diffusion pathway and fast ion transportation.

The GCD curves of NMCSs electrodes at current density of 1 A g^− 1^ are shown in Fig. 4c. The typical triangular shapes show the reversible electrochemical performance and good coulombic efficiency in the charge/discharge process. The NMCSs-600 electrode has the highest specific capacitance of 318 F g^− 1^ compared with the NMCSs-500 (280 F g^− 1^), NMCSs-700 (295 F g^− 1^) and NMCSs-800 (271 F g^− 1^). The high specific surface area allowing a great number of contact interface between the electrodes and electrolytes. While the suitable N-doped concentration (especially for N-5 and N-6 nitrogen species) leads to an improved surface wettability of the carbon materials, these can offer both of sufficient active sites and pseudocapacitance performance [32]. It explains why the NMCSs-700 has a lower specific capacitance than the NMCSs-600, although with the same specific surface area and a high O content but a lower N-doped concentration. The result suggests that the high N-doped content and the increases of the specific surface area are co-contribution to the improvement of the electrochemical capacitance. Figure 4d shows the GCD profiles of NMCSs-600 electrode at different current densities from 0.2 to 20 A g^− 1^. A good rate performance is observed and without obvious IR drop even at high current density of 20 A g^− 1^, indicating the small equivalent series resistance of the NMCSs-600 electrode [35]. However, the curves are incomplete symmetrical but slightly distorted, it can be explained by the N- and O-containing functional groups cause the combination of electric double layer capacitance and the pseudocapacitance. To detail evaluate the rate performance of the NMCSs materials, the specific capacitance of all samples calculated from the discharge curves at different current densities are presented in Fig. 4e. Apparently, the NMCSs-600 electrode has the higher specific capacitance than other NMCSs materials at the same current density. The NMCSs-600 electrode still retains a specific capacitance of 253 F g^− 1^ even at a large current density of 20 A g^− 1^, compare with the specific capacitance of 415 F g^− 1^ at 0.2 A g^− 1^, it exhibits a good capacitance retention of 61%. The electrochemical performance comparisons of the NMCSs-600 to other CSs materials synthesized by soft-template or Stöber-like methods which have reported in the literatures are summarized in Table 3. As a result, the specific capacitance of the NMCSs-600 has prominent advantages over most CSs, which is attributed to the synergetic contribution of the high pore volume, the high specific surface area and the pseudocapacitance provided by the high doped content of N and O. More importantly, the CSs synthesis time in this work is much shorter than the soft-template and Stöber-like methods in previous reported. Thus, the method reported here is a time-saving and promising strategy for preparing high performance CSs based electrodes of EDLCs.

**Table 3:** Comparison of synthesis time and electrochemical performances of CSs synthesized by soft-template or Stöber-like methods

Carbon spheres	Synthesis method	Template	Synthesis time	Specific capacitance (F g^− 1^)	Cycling	Electrolyte	Ref.
N-OMCS^a^	soft-template	F127	24 h	288 (0.1 A g^− 1^)	100% (20000)	6 M KOH	[45]
ACNS^b^	soft-template	F127	48 h	243 (0.2 A g^− 1^)	96.1% (10000)	6 M KOH	[46]
NHCSs^c^	soft-template	F127	40 h	356 (0.2 A g^− 1^)	91% (5000)	6 M KOH	[47]
NHPCNs^d^	soft-template	F127	24 h	376 (1 A g^− 1^)	95.7% (10000)	6 M KOH	[﻿37﻿]
NLEMCs^e^	soft-template	CTAC	24 h	323.2 (0.2A g^− 1^)	85% (1000)	6 M KOH	[48]
MCNs^f^	soft-template	PS-b-PEO	48 h	350 (0.1 A g^− 1^)	100% (10000)	1 M H_2_SO_4_	[49]
MCNS^g^	soft-template	F108	34 h	224 (0.2 A g^− 1^)	93% (10000)	6 M KOH	[50]
PCNS^h^	soft-template	F108	40 h	132 (0.2 A g^− 1^)	97.5% (10000)	6 M KOH	[51]
N-UCNs^i^	Stöber-like	–	48 h	269 (1 A g^− 1^)	90.3% (10000)	6 M KOH	[25]
MCSs^j^	Stöber-like	–	48 h	196 (1 mV s^− 1^)	–	1 M H_2_SO_4_	[52]
MCMs^k^	Stöber-like	SiO_2_	48 h	289 (1 A g^− 1^)	90.3% (10000)	6 M KOH	[53]
MMCSs^l^	Stöber-like	SiO_2_	48 h	314 (0.5 A g^− 1^)	96% (500)	6 M KOH	[54]
NMCSs	Stöber-like	F108	6 h	415 (0.2 A g^− 1^) 357 (0.5 A g^− 1^)	96.9% (10000)	6 M KOH	This work

^a^N-doped ordered mesoporous CSs

^b^Activated carbon nanospheres

^c^N-doped hierarchical CSs

^d^N-doped hierarchical porous carbon nanospheres

^e^N-doped lychee exocarp-like mesoporous CSs

^f^Mesoporous carbon nanospheres

^g^Monodisperse carbon nanospheres

^h^Porous carbon nanospheres

^I^N-containing ultramicroporous carbon nanospheres

^j^Monodisperse CSs

^k^Mesoporous carbon microspheres

^l^Micro- and mesoporous CSs

The cycle life of electrode materials is definitely essential parameter during the practical application process of energy storage and conversion devices. The long-term cycling stability of the NMCSs-600 electrode is evaluated by the charge/discharge cycling at a current density of 10 A g^− 1^. As shown in Fig. 4f, the specific capacitance retention is 96.9% of the initial capacitance after 10,000 cycles, suggesting the NMCSs-600 material has a superior cycle stability performance. In more detail, the almost similar GCD curves of the first five and last five charge/discharge cycles also confirm the reversible process and cycling stability (inset of Fig. 4f). The unique structural advantages of microporous CSs endow the excellent cycle stability and coupled with the high specific capacitance demonstrate a great potential as promising electrode materials for supercapacitors.

**Fig. 5:**
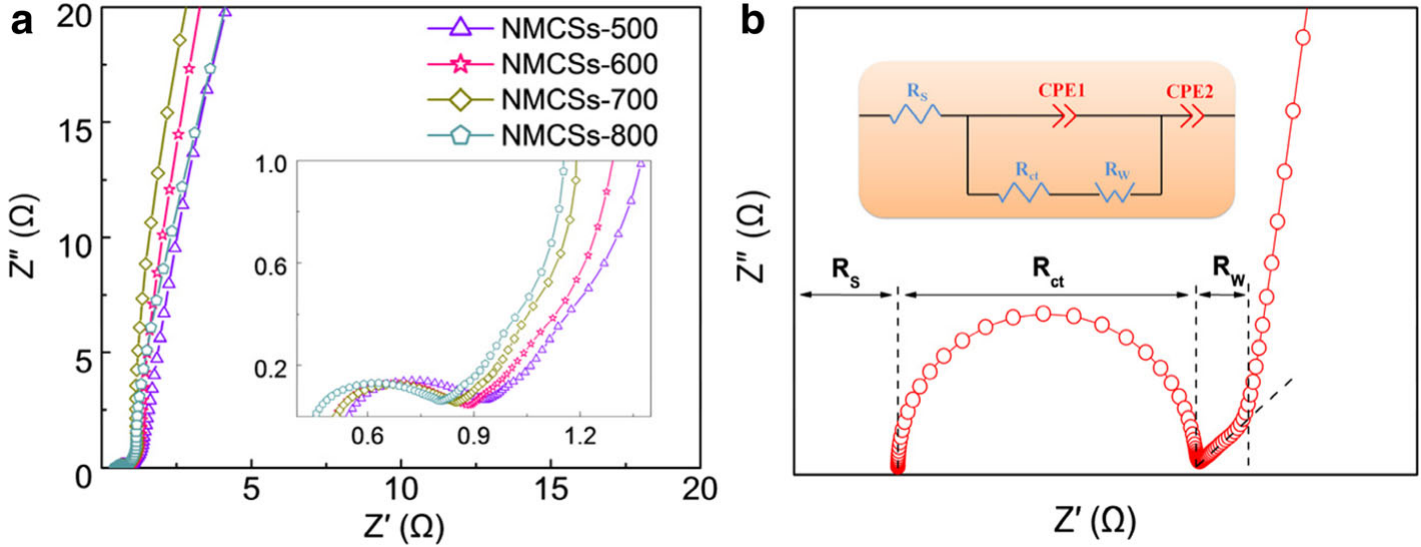
(**a**) Nyquist plots of NMCSs materials and the inset shows the magnify plots at high frequency range and (**b**) a typical Nyquist plot of EDLCs and the equivalent circuit model

EIS is a powerful method to study the charge transport information and the kinetics process in the electrode/electrolyte interface, such as capacitance characteristic, resistance property and ion migration behaviors [﻿36﻿]. The electrochemical properties of as-prepared materials are explored by EIS measurement. Figure 5a shows the Nyquist plots of NMCSs electrodes in a frequency range from 0.01 Hz to 10 kHz. It can be seen that the curves of all samples are very similar shape, which like a typical Nyquist plot of EDLCs presented in Fig. 5b. The first intersection point on the real *Z* axis refers to the equivalent series resistance (*R*_S_), which mainly comprises the intrinsic resistance of the electrode materials, the electrolyte resistance and the contact resistance of the electrode/current collector [2]. The diameter of quasi-semicircle presence at the high frequency reflects the charge transfer resistance (*R*_ct_) in electrodes/electrolyte interface. A nearly 45° straight line in the intermediate frequency denotes the Warburg impedance (*R*_w_), representing the diffusion transportation rate of electrolyte ions in the pore channel of carbon materials [7]. In the low frequency region all samples exist an almost vertical line suggests that the NMCSs materials have an ideal capacitive performance and without diffusion limit in the electrode. The equivalent circuit model is shown in the inset of Fig. 5b, and the various resistances fitting data of NMCSs electrodes are listed in Additional file 1: Table S1. All samples have small equivalent series resistance and semicircle diameter indicate a good electrical conductivity and contact interface, which could be due to the high N-doped concentration improving the electronic character and wettability of those carbon materials. Furthermore, the short Warburg-type line reveals that appropriate porosity matching perfect with the electrolyte ions and minimize the diffusion resistance for mass transport at the pore channels.

### Electrochemical Performance of the NMCSs-600-Based SSDs

**Fig. 6:**
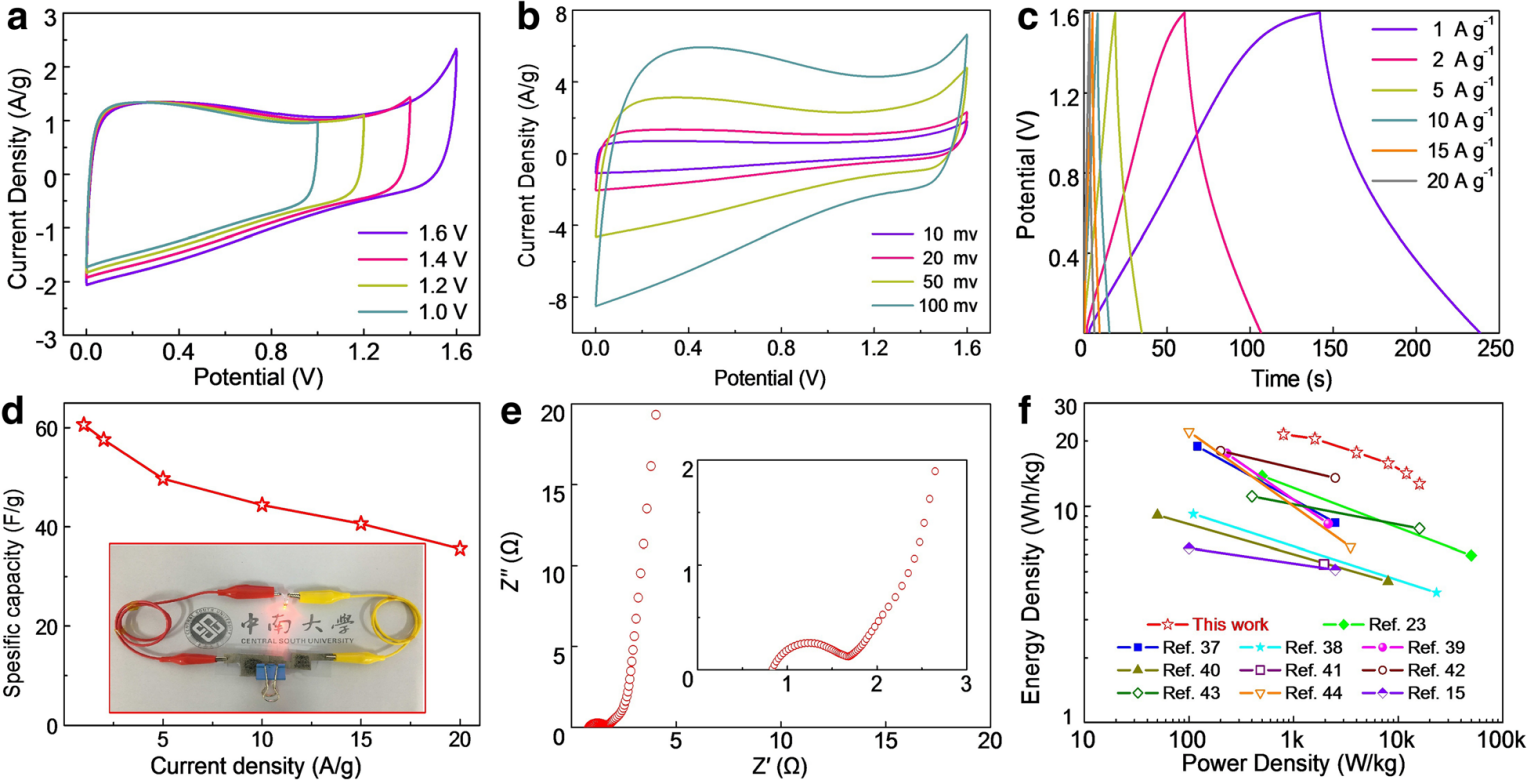
The electrochemical characteristics of the assembled SSDs based on the NMCSs-600 materials using PVA/KOH as the gel electrolyte in two electrode system. **a** CV curves of the SSD in different voltage windows from 1 to 1.6 V at the scan rate of 20 mV s^− 1^. **b** CV curves of the SSD at various scan rates within a voltage window of 1.6 V. **c** GCD curves at different current densities. **d** The gravimetric capacitance of the SSD as a function of current density, the inset image shows a commercial red LED powered by two SSDs in series. **e** Nyquist plot of the SSD, the inset gives the magnify plot for high frequency range. **f** Ragone plots of the SSD and the other carbon spheres based symmetric supercapacitors

In order to demonstrate the practical applications of the as-prepared NMCSs-600 materials, the SSDs are assembled by the identical NMCSs-600 electrodes and the gel electrolyte of PVA/KOH. The electrochemical performances of NMCSs-600-based SSDs are evaluated by two-electrode system. To determine the maximum voltage window, Fig. 6a show the CV curves of the NMCSs-600-based SSD measurement at scan rate of 20 mV s^− 1^ with different voltage windows range from 1 V to 1.6 V. The CV curves exhibit a rectangular-like shape in the work windows from 1 to 1.4 V, indicating the ideal EDLCs behavior. When the voltage window increases to 1.6 V, a slightly anodic current polarization peak begins to appear. Thus, 1.6 V is selected as the work voltage window to study the electrochemical performances of the SSDs. Figure 6b shows the CV curves of the SSD at different scan rates from 10 to 100 mV s^− 1^ over a voltage window of 1.6 V. Obviously, the current density increasing with the scan rate, and a quasi-rectangular shape is well maintains even at a high scan rate of 100 mV s^− 1^. It suggests that the as-prepared SSD has ideal supercapacitor behavior and fast charge transportation. In addition, the SSD presents a wide and reversible peak at 0.4 V with a little distort, demonstrating the good pseudocapacitance performance provided by N- and O-doped. Moreover, the GCD curves of the SSD are also performed at various current densities from 1 to 20 A g^− 1^ (Fig. 6c). As expected, the nearly triangular shape can be observed, showing it is a reversible charge/discharge process. The specific capacitance of the NMCSs-600-based SSD as a function of current density is shown in Fig. 6d. A maximum capacitance of 60.6 F g^− 1^ can be reached at current density of 1 A g^− 1^ and retains 37.5 F g^− 1^ at 20 A g^− 1^, demonstrate the good rate performance and high capacitance retention. EIS measurement is conducted to investigate the interface contact and electrochemical performance of the SSDs. According to the Nyquist plot (Fig. 6e), a small equivalent series resistance of 0.83 Ω and charge transfer resistance of 0.85 Ω are obtained, manifesting the excellent electronic conductivity of the as-prepared SSD and good interface contact between the NMCSs-600 electrodes and the PVA/KOH electrolyte. In addition, the low Warburg resistance of 0.52 Ω and a nearly straight line at low frequency reveal the fast charge transportation as well as ion diffusion, which represent a favorable capacitive performance of the NMCSs-600-based SSDs. In addition, the NMCSs-600-based SSD displays good cycling stability with 80% retention after 2000 consecutive cycles at a current density of 10 A g^− 1^(Additional file 1: Figure S4).

Energy density and power density are two key parameters for assess the practical applications of supercapacitor devices. The Ragone plot displayed in Fig. 6f shows the NMCSs-600-based SSD exists a maximum energy density of 21.5 Wh kg^− 1^ at a power density of 800 W kg^− 1^ and the energy density still maintains 13.3 Wh kg^− 1^ even at a power density as high as 16 kW kg^− 1^. As shown in Fig. 6f and Additional file 1: Table S2, the NMCSs-600-based SSD has a great advantages compared with other CSs based supercapacitor devices, such as core-shell ultramicroporous@microporous carbon nanospheres [23], N-doped carbon nanospheres [﻿37﻿–39], N and O co-doped carbon microspheres [﻿40﻿], hollow CSs [﻿41﻿], graphitic hollow CSs [﻿42﻿], N-doped hollow CSs [﻿43﻿, ﻿44﻿] and nitrogen-phosphorus co-doped hollow carbon microspheres [15]. Furthermore, two as-fabricated NMCSs-600-based SSDs are connected in series could power a red light emitting diode (inset of Fig. 6d), and the light intensity without obvious decrease after 60 s (as shown in Video S1). Therefore, all those impressive electrochemical performances show attractive potential applications of the NMCSs-600-based SSD for energy storage.

## Conclusions

In summary, NMCSs have been successfully prepared through a simple one-pot and time-saving one-step hydrothermal polymerizing of PF resin in the existence of F108 used as a soft-template, subsequent by carbonization and KOH activation. The high concentration ammonia and high hydrothermal temperature accelerated the polymerization process and caused the short reaction time for 6 h. In the hydrothermal process, ammonia was not only as a catalyst, but also served as a nitrogen source to introduce the N-heteroatom into the CSs framework which makes a high N-doped content of 2.6 at.%. The optimized NMCSs with the ethanol/water volume ratio of 1:1 were exhibited smooth surface, perfect spherical morphology and good dispersity. At optimal carbonization temperature of 600 °C, the NMCSs-600 have the highest specific surface area of 1517 m^2^ g^− 1^ with the largest total pore volume of 0.8 cm^3^ g^− 1^, which offered enough electrode/electrolyte contact interface and abundant active sites. The unique structural advantages of microporous CSs and appropriate porosity matched perfectly with the electrolyte ions were endowed fast transportation of ions in the pore channels. As a result, as supercapacitor electrodes, the as-prepared NMCSs-600 material have shown an outstanding specific capacitance of 416 F g^− 1^ at a current density of 0.2 A g^− 1^ (357 F g^− 1^ at 0.5 A g^− 1^) and excellent charge/discharge cycling stability with 96.9% capacitance retention after 10,000 cycles. Furthermore, the constructed NMCSs-600-based SSD has shown a high specific capacitance of 60.6 F g^− 1^ at current density of 1 A g^− 1^, a maximum energy density of 21.5 Wh kg^− 1^ has been achieved at a power density of 800 W kg^− 1^ and the energy density still maintained 13.3 Wh kg^− 1^ even at a high power density of 16 kW kg^− 1^. Therefore, the time-saving and effective synthesis strategy coupled with the remarkable electrochemical performances may create a new situation for developing high energy density and high power density of energy storage and conversion devices.

## Additional file

Additional file 1: **Figure S1.** The assemble process of NMCSs-600-based symmetric supercapacitors. **Figure S2.** The high-resolution C 1 s spectra of the as-prepared NMCSs materials at different carbonization temperatures of (a) 500 °C, (b) 600 °C, (c) 700 °C and (d) 800 °C. **Figure S3.** The high-resolution O 1 s spectra of the as-prepared NMCSs materials at different carbonization temperatures of (a) 500 °C, (b) 600 °C, (c) 700 °C and (d) 800 °C. **Figure S4.** The cycling performance of the NMCSs-600-based SSD at current density of 10 A g− 1 for 2000 cycles. **Table S1.** The different resistance values of the NMCSs samples. **Table S2.** Comparison of energy density and power density data reported for different CSs based symmetric supercapacitor devices. (DOCX 752 kb)

## Abbreviations

NMCSs: Nitrogen-doped microporous carbon spheres
EDLCs: Electrical double-layer capacitors
CSs: Carbon spheres
SSDs: Symmetric supercapacitor devices
CV: Cyclic voltammetry
GCD: Galvanostatic charge/discharge
EIS: Electrochemical impedance spectroscopy
