# Supplementary material for: Facile Synthesis of Nitrogen-Doped Microporous Carbon Spheres for High Performance Symmetric Supercapacitors
Source: Nanoscale Res Lett. 2018 Oct 4;13:314. doi: 10.1186/s11671-018-2713-0 (PMC6172159; doi:10.1186/s11671-018-2713-0)
Supplement: Supplementary file 1 — Figure S1. The assemble process of NMCSs-600-based symmetric supercapacitors. Figure S2. The high-resolution C 1 s spectra of the as-prepared NMCSs materials at different carbonization temperatures of (a) 500 °C, (b) 600 °C, (c) 700 °C and (d) 800 °C. Figure S3. The high-resolution O 1 s spectra of the as-prepared NMCSs materials at different carbonization temperatures of (a) 500 °C, (b) 600 °C, (c) 700 °C and (d) 800 °C. Figure S4. The cycling performance of the NMCSs-600-based SSD at current density of 10 A g− 1 for 2000 cycles. Table S1. The different resistance values of the NMCSs samples. Table S2. Comparison of energy density and power density data reported for different CSs based symmetric supercapacitor devices. (DOCX 752 kb) [file 11671_2018_2713_MOESM1_ESM.docx]

**Supplementary Information**

**Facile Synthesis of Nitrogen-doped Microporous Carbon Spheres for High Performance Symmetric Supercapacitors**

Zhongguan Liang^1^, Hao Liu^1^, Jianping Zeng^2^, Jianfei Zhou^1^, Hongjian Li^1^ and Hui Xia^1^*

^1^School of Physics and Electronics, Central South University, Changsha 410083, China.

^2^School of Physics and Electronics, Hunan University, Changsha 410082, China.

*Corresponding author. E-mail: [xhui73@csu.edu.cn](mailto:xhui73@csu.edu.cn) (H. Xia).

**PACS:** 81.05.Uw; 88.80.Fh; 82.47.Uv


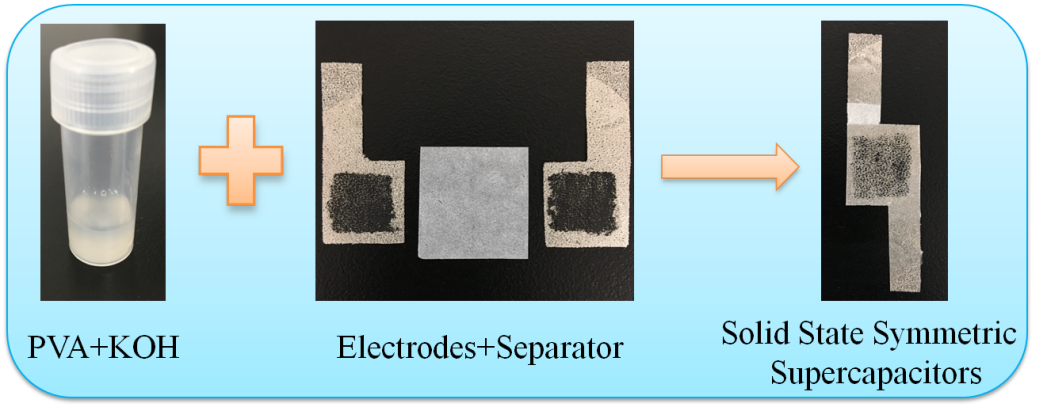


**Figure S1** The assemble process of NMCSs-600-based symmetric supercapacitors.

**Figure S2** The high-resolution C 1s spectra of the as-prepared NMCSs materials at different carbonization temperatures of (a) 500℃, (b) 600℃, (c) 700℃ and (d) 800℃.

**Figure S3** The high-resolution O 1s spectra of the as-prepared NMCSs materials at different carbonization temperatures of (a) 500℃, (b) 600℃, (c) 700℃ and (d) 800℃.

**
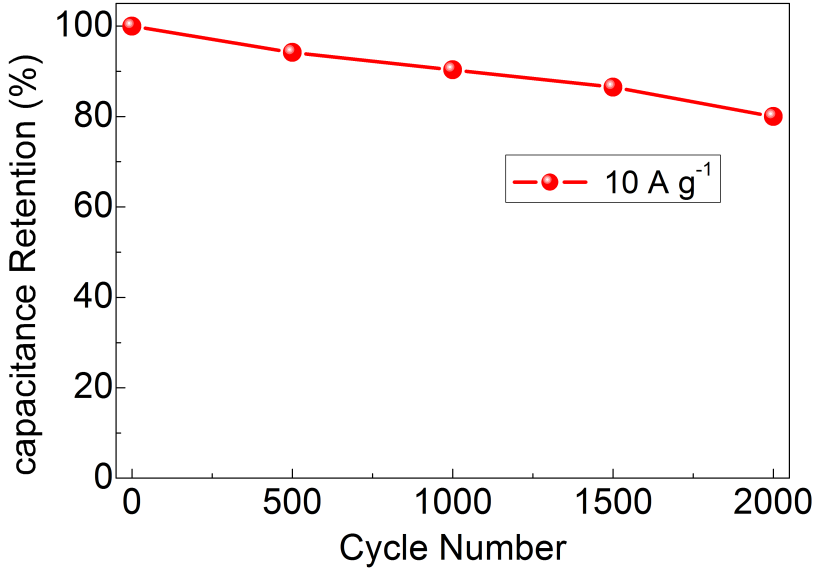
**

**Figure S4** The cycling performance of the NMCSs-600-based SSD at current density of 10 A g^-1^ for 2000 cycles.

**Table S1** The different resistance values of the NMCSs samples

| Samples | *R*_S_ (Ω) | *R*_ct_ (Ω) | *R*_W_ (Ω) |
| --- | --- | --- | --- |
| NMCSs -500 | 0.55 | 0.38 | 0.30 |
| NMCSs -600 | 0.51 | 0.37 | 0.28 |
| NMCSs -700 | 0.51 | 0.34 | 0.28 |
| NMCSs -800 | 0.45 | 0.36 | 0.27 |

**Table S2** Comparison of energy density and power density data reported for different CSs based symmetric supercapacitor devices

| Carbon material | Device type | Electrolyte | Energy density (Wh/kg) | Power density (kW/kg) | Ref. |
| --- | --- | --- | --- | --- | --- |
| UMCNs^a^ | symmetric | 6M KOH | 13.8  5.94 | 0.5  50 | [[1](#_ENREF_1)] |
| NHPCNs^b^ | symmetric | 1M TEABF_4_/AN | 18.9  8.4 | 0.12  2.5 | [[2](#_ENREF_2)] |
| NRMC^c^ | symmetric | 6M KOH | 9.2  4 | 0.11  23.24 | [[3](#_ENREF_3)] |
| C-PANI^d^ | symmetric | 0.5M Na_2_SO_4_ | 17.5  8.3 | 0.23  2.15 | [[4](#_ENREF_4)] |
| NO-MMCNs^e^ | symmetric | 6M KOH | 9.1  4.5 | 0.05  8 | [[5](#_ENREF_5)] |
| HCSs^f^ | symmetric | 6M KOH | 5.4 | 1.95 | [[6](#_ENREF_6)] |
| GHCSs^g^ | symmetric | 1M Na_2_SO_4_ | 2.6 | 3.0 | [[7](#_ENREF_7)] |
| N-HMCSs^h^  N-YSHMCSs^i^ | symmetric | 1M H_2_SO_4_ | 7.9  5.5 | 16  16 | [[8](#_ENREF_8)] |
| NPHCMs^j^ | symmetric | PVA/KOH | 6.4  5.1 | 0.1  50 | [[9](#_ENREF_9)] |
| NMCSs | symmetric | PVA/KOH | 21.5  13.3 | 0.8  16 | This work |

^a^Core-shell ultramicroporous@microporous carbon nanospheres, ^b^N-doped hierarchical porous carbon nanospheres, ^c^N-doped carbon nanospheres, ^d^N-containing polyaniline-based carbon nanospheres, ^e^N- and O-containing micro-mesoporous carbon microspheres, ^f^Hollow microporous CSs, ^g^Graphitic hollow CSs, ^h^N-doped hollow mesoporous carbon nanospheres, ^i^Yolk−shell hollow mesoporous carbon nanospheres, ^j^Nitrogen-phosphorus co-doped hierarchical porous carbon microspheres.

**References**

[1] Liu M, Qian J, Zhao Y, Zhu D, Gan L, Chen L (2015) Core-shell ultramicroporous@microporous carbon nanospheres as advanced supercapacitor electrodes. J Mater Chem A 3(21):11517-11526

[2] Xiong S, Fan J, Wang Y, Zhu J, Yu J, Hu Z (2017) A facile template approach to nitrogen-doped hierarchical porous carbon nanospheres from polydopamine for high-performance supercapacitors. J Mater Chem A 5(34):18242-18252

[3] Sun F, Gao J, Pi X, Wang L, Yang Y, Qu Z, Wu S (2017) High performance aqueous supercapacitor based on highly nitrogen-doped carbon nanospheres with unimodal mesoporosity. J Power Sources 337:189-196

[4] Peng H, Ma G, Sun K, Mu J, Zhou X, Lei Z (2015) A novel fabrication of nitrogen-containing carbon nanospheres with high rate capability as electrode materials for supercapacitors. RSC Adv 5(16):12034-12042

[5] Liu S, Chen X, Li X, Huo P, Wang Y, Bai L, Zhang W, Niu M, Li Z (2016) Nitrogen- and oxygen-containing micro-mesoporous carbon microspheres derived from m-aminophenol formaldehyde resin for supercapacitors with high rate performance. RSC Adv 6(92):89744-89756

[6] Wang K, Huang L, Razzaque S, Jin S, Tan B (2016) Fabrication of hollow microporous carbon spheres from hyper-crosslinked microporous polymers. Small 12(23):3134-3142

[7] Lei Z, Zhang J, Zhao XS (2012) Ultrathin MnO_2_ nanofibers grown on graphitic carbon spheres as high-performance asymmetric supercapacitor electrodes. J Mater Chem 22(1):153-160

[8] Liu C, Wang J, Li J, Zeng M, Luo R, Shen J, Sun X, Han W, Wang L (2016) Synthesis of N-doped hollow-structured mesoporous carbon nanospheres for high-performance supercapacitors. ACS Appl Mater Interfaces 8(11):7194-7204

[9] Zhang N, Liu F, Xu S, Wang F, Yu Q, Liu L (2017) Nitrogen-phosphorus co-doped hollow carbon microspheres with hierarchical micro-meso-macroporous shells as efficient electrodes for supercapacitors. J Mater Chem A 5(43):22631-22640
